# APP Deletion Accounts for Age-Dependent Changes in the Bioenergetic Metabolism and in Hyperphosphorylated CaMKII at Stimulated Hippocampal Presynaptic Active Zones

**DOI:** 10.3389/fnsyn.2017.00001

**Published:** 2017-01-20

**Authors:** Melanie Laßek, Jens Weingarten, Martin Wegner, Moritz Neupärtl, Tabiwang N. Array, Eva Harde, Benedikt Beckert, Vahid Golghalyani, Jörg Ackermann, Ina Koch, Ulrike C. Müller, Michael Karas, Amparo Acker-Palmer, Walter Volknandt

**Affiliations:** ^1^Institute for Cell Biology and Neuroscience, Biologicum and BMLS, Johann Wolfgang Goethe-UniversitätFrankfurt, Germany; ^2^Molecular Bioinformatics, Johann Wolfgang Goethe-UniversitätFrankfurt, Germany; ^3^Institute of Pharmaceutical Chemistry, Johann Wolfgang Goethe-UniversitätFrankfurt, Germany; ^4^Thermo Fisher ScientificBremen, Germany; ^5^Max Planck Institute for Brain ResearchFrankfurt, Germany; ^6^Department of Pharmacy and Molecular Biotechnology, University HeidelbergHeidelberg, Germany

**Keywords:** aging, amyloid precursor protein, hippocampus, LTP, mitochondria, presynaptic active zone

## Abstract

Synaptic release sites are characterized by exocytosis-competent synaptic vesicles tightly anchored to the presynaptic active zone (PAZ) whose proteome orchestrates the fast signaling events involved in synaptic vesicle cycle and plasticity. Allocation of the amyloid precursor protein (APP) to the PAZ proteome implicated a functional impact of APP in neuronal communication. In this study, we combined state-of-the-art proteomics, electrophysiology and bioinformatics to address protein abundance and functional changes at the native hippocampal PAZ in young and old APP-KO mice. We evaluated if APP deletion has an impact on the metabolic activity of presynaptic mitochondria. Furthermore, we quantified differences in the phosphorylation status after long-term-potentiation (LTP) induction at the purified native PAZ. We observed an increase in the phosphorylation of the signaling enzyme calmodulin-dependent kinase II (CaMKII) only in old APP-KO mice. During aging APP deletion is accompanied by a severe decrease in metabolic activity and hyperphosphorylation of CaMKII. This attributes an essential functional role to APP at hippocampal PAZ and putative molecular mechanisms underlying the age-dependent impairments in learning and memory in APP-KO mice.

## Introduction

Almost 30 years ago, the amyloid precursor protein (APP) was discovered as precursor of amyloid-beta-peptide (Aβ), one of the main constituents of senile plaques (Kang et al., 1987). Deposits of senile plaques and accumulation of neurofibrillary tangles are hallmarks of Alzheimer’s Disease (AD) the most common form of dementia. The pathophysiology of AD is basically characterized by a massive loss of synapses, cognitive decline and behavioral changes attributed to the accumulation of Aβ especially in the hippocampus (Iqbal et al., 1986; Leuner et al., 2012a). During the last decade, much effort was pursued in understanding the pathology of AD and to develop therapeutic strategies. However, the mechanisms accounting for the accumulation of Aβ and the degeneration of neurons have not yet been identified. It is widely accepted, that APP acts as a key protein in AD, but its physiological function is rather unknown. In the CNS APP is a neuron-specific protein and is localized at the presynaptic active zone (PAZ; Guo et al., 2012; Müller and Zheng, 2012; Laßek et al., 2013). Embedding APP into the evolutionary conserved active zone protein network highlighted APP as a context-sensitive regulator of the hippocampal PAZ, indicating its crucial role in the synaptic vesicle cycle, Ca^2+^-homeostasis and cytoskeletal rearrangement (Laßek et al., 2016).

The generation of genetically modified mouse models further identified APP as an essential player in synapse formation and plasticity (Heber et al., 2000; Wang et al., 2005, 2009; Ring et al., 2007; Weyer et al., 2011, 2014; Hick et al., 2015). The phenotype of APP deletion is age-dependent: young mice reveal no physiological alteration, except from an increase in neuron numbers within the hippocampal stratum radiatum and stratum moleculare. This increase in hippocampal synapses was not preserved in the elderly mice (Priller et al., 2006).

Upon aging, APP-KO accounts for severe impairments in learning and memory associated with deficits in long-term-potentiation (LTP; Ring et al., 2007). Interestingly, reduced dendritic length and branching of CA1 pyramidal neurons accompanied by a reduction in total spine density was characteristic for old APP-KO mice and indicates a physiological role of APP in maintaining spine density structure (Lee et al., 2010; Tyan et al., 2012). At protein level, the calcium and calmodulin-dependent kinase II (CaMKII) plays a prominent role in the setup of learning and memory. The enzyme is discussed to be responsible for general changes in synaptic efficacy, after Ca^2+^-influx and moreover, to be required for proper LTP-induction in the hippocampus (Rongo, 2002).

The hippocampus, central for learning and memory consolidation, receives input from the entorhinal cortex, the main interface between hippocampus and neocortex. Neurons from layer II of the perforant path project to the dentate gyrus granule cells and CA3 pyramidal cells (CA, cornu ammonis). Neurons from layer III project to the pyramidal cells of the CA1 region and the subiculum via the temporoammonic pathway (Neves et al., 2008; Deng et al., 2010). In the 1970s, LTP was discovered by T. Bliss and T. Lømo. The experimental establishment of LTP within the hippocampus serves as a suitable model of activity-dependent synaptic plasticity (Bliss and Collingridge, 1993). There are two LTP-forms to be distinguished: associative LTP (perforant pathway) and non-associative LTP (mossy fiber pathway; Bliss and Collingridge, 1993). In general, LTP can experimentally be induced by a strong synaptic stimulus, either electrically (theta burst or high frequency stimulation) or chemically, e.g., application of the K^+^-channel blocker tetraethylammonium (TEA; Song et al., 2002). In both cases, presynaptic L-type voltage-gated calcium channels (VGCCs) and postsynaptic N-Methyl-D-aspartate receptors (NMDARs) are required (Zakharenko et al., 2001). Interestingly, APP is involved in the recruitment and regulation of VGCC at hippocampal neurotransmitter release sites and APP-KO mice reveal diminished endocytosis of VGCC accompanied by a dysregulation in the balance between inhibitory and excitatory neurons. The increase in abundance of presynaptic VGCC resulted in increased activity of GABAergic neuronal activity (Yang et al., 2009). In this context, the impairment in learning and memory in APPsα-KI/APLP2-KO mice could be rescued by the application of the GABA_A_ receptor inhibitor picrotoxin (Weyer et al., 2011). The phenotype observed for APP-mutants indicates a critical role of APP in memory formation and consolidation, as well as in behavioral aspects. Moreover, a yet unknown compensatory mechanism executed by APP family members is suggested for the mild APP-KO phenotype (reviewed in Müller and Zheng, 2012).

At neurotransmitter release sites, mitochondria are essential for providing energy supply and calcium buffering for a multitude of physiological functions. Besides the glycolytic chain associated with synaptic vesicles (reviewed in Burré and Volknandt, 2007), mitochondria are the main source for the production of ATP at the presynaptic terminal. Therefore, mitochondria are essential in maintaining presynaptic homeostasis and phosphorylation reactions and are highly involved in synaptic plasticity (reviewed in Mattson et al., 2008). The mitochondrial machinery for energy production is the electron transport chain (ETC), consisting of five complexes and organized in an assembly line-like manner across the inner membrane. The proton gradient necessary for the ATP production at complex V (ATP-synthase) is generated by complex I, II and IV (see Figure 4). Complex I and complex III are the main sources of reactive oxygen species (ROS) production that accounts for mitochondrial dysfunction upon aging and in neurodegenerative diseases (Leuner et al., 2012b). Modulators of energy production and ROS production are coenzyme Q_10_ at complex III and cytochrome *c* at complex IV (reviewed in Mattson et al., 2008). Under physiological conditions, the energy-transducing capacity of mitochondria meets the energy demand of neurons, supporting crucial metabolic and mechanical functions (Yin et al., 2014). It is widely accepted that changes in the homeostasis between APP, Aβ and mitochondrial function are likely to correlate with the onset of neurodegenerative diseases e.g., AD (Eckert et al., 2012; Leuner et al., 2012a). The early onset of AD reveals a decrease in brain metabolism and increase in oxidative stress long before the deposition of Aβ-plaques or tau-fibrils. In this context, age-dependent alterations in bioenergetic metabolism accompanied by changes in the ETC are likely to contribute to disturbed neuronal function (Yao et al., 2009; Yin et al., 2014).

Within the scope of this article, we demonstrate that APP deletion accounts for age-dependent changes in bioenergetic metabolism and hyperphosphorylation of CaMKII at stimulated hippocampal PAZs. These alterations at hippocampal neurotransmitter release sites may provide a missing link by unraveling the age-dependent impairments in learning and memory.

## Materials and Methods

### Animals

Animal treatment was performed under veterinary supervision in accordance with animal welfare regulations of the German animal protection law (Regierungspräsidium Darmstadt and Karlsruhe, Darmstadt, Germany). All mice were maintained in an animal facility, and the experimental procedures were approved by the Animal Welfare office of the Regierungspräsidium Darmstadt and Karlsruhe. APP-KO (APP^−/−^) backcrossed to C57BL/6N mice were compared to wildtype (wt; C57BL/6N). All groups were age-matched (4 and 24 months, respectively). Mice were kept under 12 h light and dark cycle with food and water *ad libitum*. The generation of APP mutant mice has previously been described (Li et al., 1996).

### Antibodies

SV2 (clone CKK 10H4 producing the monoclonal anti-SV2 antibody, kindly donated by Dr. Regis B. Kelly, San Francisco, CA, USA); was cultured in-house.

Dynabeads M-280 conjugated with monoclonal sheep anti-mouse IgGs (cat. No. 112.02D) were purchased from Invitrogen, Darmstadt, Germany.

### Subcellular Fractionation of the Hippocampal PAZ

The hippocampus was dissected from native mouse brain prior to subcellular fractionation. Synaptic vesicles were isolated from synaptosomes according to the protocol guidelines of Whittaker (Whittaker et al., 1964). The protocol has previously been adapted to the fractionation of individual mouse brains (Weingarten et al., 2014) and was downscaled for individual mouse brain regions (Weingarten et al., 2015). The following modifications were applied: individual hippocampi were homogenized in 0.4 mL of preparation buffer (5 mM Tris-HCl, 320 mM sucrose, pH 7.4) containing the protease inhibitors antipain, leupeptin, chymostatin (2 μg/mL each), pepstatin (1 μg/mL) and benzamidine (1 mM). Unless otherwise mentioned the material was kept at 4°C during the entire preparation. The hippocampal homogenate was centrifuged using a Beckman TLX Optima 120 and rotor TLA 120.2 by acceleration (mode 4) up to 2800_gav_ for 2 min. The resulting pellet was discarded and the supernatant was further fractionated by discontinuous Percoll gradient centrifugation. The Percoll gradient was prepared by layering 1.0 mL supernatant solution onto three layers of 1.0 mL Percoll solution (3%, 10%, 23% (v/v) in preparation buffer). After centrifugation using the TLA 100.4 rotor for 7 min at 35,000_gav_, fractions containing synaptosomes were collected and diluted twofold in preparation buffer and centrifuged using TLA 100.4 rotor for 35 min at 50,000_gav_. For hypoosmotic lysis of synaptosomes the resulting pellet was triturated in four volumes of lysis buffer (5 mM Tris-HCl, pH 7.4) at room temperature. The suspension was centrifuged using the TLA 100.4 rotor for 60 min at 250,000_gav_. The pellet was resuspended and homogenized in 300 μL sucrose buffer (10 mM HEPES-NaOH, 0.5 mM EGTA, 0.1 mM MgCl_2_, 200 mM sucrose, pH 7.4). This microsomal solution was layered onto 900 μL of a discontinuous sucrose gradient (0.3 M, 0.75 M, and 1.2 M; containing 10 mM HEPES, 0.5 mM EGTA, adjusted to pH 7.4) and centrifuged using a WX Ultra 90 Sorvall centrifuge and the TST 55.5 rotor for 2 h at 65, 000_gav_. Thirty-six fractions (35 μL each) were collected from top to bottom of the gradient. The pooled lower fractions (LF) 16–30 corresponding to sucrose concentrations of 0.5–1.1 M were further analyzed.

### Immunopurification of the Hippocampal Presynaptic Active Zone via Docked Synaptic Vesicles

The Immunopurification (IP) protocol for the PAZ via docked synaptic vesicles was as described recently for individual mouse brain regions (Weingarten et al., 2015). In brief, 100 μL magnetic beads pre-coupled with an anti-mouse monoclonal antibody were washed with Tris-buffered saline (TBS, pH 7.4) and incubated with TBS containing 1% glycine, 1% lysine and 0.5% saponin followed by three washing steps in TBS. Magnetic beads were then incubated for 1 h with the anti-SV2 antibody (3 μg of antibody per 10^7^ magnetic beads to gain representative SV2 population). Crosslinking of the antibodies was performed with 0.1% glutardialdehyde in TBS for 5 min and stopped by adding TBS containing 1% glycine and 1% lysine. Finally the beads were incubated over night at 4°C with the pooled lower sucrose gradient fractions (LF, 16–30). Beads containing the bound material were washed three times with TBS and incubated with ice-cold acidified acetone (acetone containing 125 mM HCl) for 30 min at 20°C. Elution was performed with different elution agents for 30 min. For Western blot analysis proteins were eluted with sample buffer containing 2% SDS. For MS analysis proteins were eluted with 100 mM triethylammonium bicarbonate (TEAB). The elution of PAZ proteins was supported by applying short ultrasonic pulses.

### Mass Spectrometry—LC-MS/MS Analysis of Individual Hippocampal PAZ

The immunopurified PAZ derived from mouse hippocampus was subjected to enzymatic digestion using the well-established serine protease trypsin. The amount of trypsin (Proteomics Grade, Sigma Aldrich, St. Louis, MO, USA) was adjusted to an enzyme-to-substrate ratio of 1:50 for each sample according to the protein concentrations determined by BCA protein assay (Pierce, Thermo Scientific, Rockford, IL, USA). The digestion of equal amounts of purified protein was performed at 37°C for 18 h and stopped by acidification with 10% formic acid (FA) solution. Samples were dried in a vacuum concentrator.

Peptides were labeled with Tandem Mass Tag^TM^ 6-plex (TMTsixplex^TM^) reagents (Thermo Scientific^TM^, Rockford, IL, USA) according to the manufacturer’s protocol. The immunopurified hippocampal PAZ peptides derived from the respective wild type mice was labeled with TMT-126, TMT-127 and TMT-128, whereas the immunopurified hippocampal PAZ from APP mutants was labeled with TMT-129, TMT-130, and TMT-131. The peptide mixtures were combined in equal quantity, vacuum-dried, purified and desalted by employing Pierce C18 spin columns (Thermo Scientific, Rockford, IL, USA) according to the User’s Guide and finally vacuum-dried. Prior to separation the sample was solubilized in 10 μl solvent C (0.1% FA, 2% MeCN).

Chromatographic separation of the mixture was performed on an EASY-nLC^TM^ 1000 System (Thermo Scientific, Rockford, IL, USA). Peptides were separated on an Acclaim PepMap100 (75 μm i.d. × 50 cm, packed with C18, 2 μm particles, 100 Åpore size) EASY-Spray nano column at a flow rate of 250 nl/min. A TMT-peptide adapted gradient of 148 min with increasing amounts of solvent B (0.1% FA in MeCN) was applied. The LC system was coupled online to a Q-Exactive HF hybrid quadrupole-Orbitrap instrument (Thermo Scientific) and operated in a data-dependent acquisition mode selecting the top 15 most intense peaks from a survey scan for fragmentation.

The survey scan was performed using the following parameters: scan range between 380–1400 m/z at a resolute ion of 120,000 at m/z 200. The AGC target value was set to 3 + e6. MS/MS scans were acquired at a resolution of 30,000 at m/z 200 with an AGC target of 1 + e5. The precursors were isolated with an isolation width of 1.2 m/z and the normalized collision energy (NCE) was 32. Dynamic exclusion was set to 20 s, peptide recognition mode was enabled while only charge states of 2–6 were considered for fragmentation and unassigned precursor ions were disabled. Spectra were deconvoluted by the integrated software module and a signal-to-noise filter of 1.5 was applied. Data processing, database searches for protein identification and relative quantification were performed using an in-house Mascot (Version 2.4) server. Precursor mass tolerance was set to 5 ppm and fragment mass tolerance to 0.1 Da. Oxidation of methionine was allowed as variable modifications and TMT was set as fixed modification for lysine residues and peptide N-termini. Spectra were searched against a database of murine proteins (SwissProt, released on 2014-02-19) and a decoy (reversed DB) search was performed, with a target FDR value of ≤1.5%. For protein quantification by weighted average only unique peptides were considered and the peptide ratios were normalized by “average ratio”. Only proteins that were identified in each experiment were considered for further analysis, and defined as core proteome.

### Phosphopeptide Analysis

Peptides identified by MS/MS were analyzed for their phosphorylation status. Our data sets were searched against a self-established database of the core proteome deduced from a murine protein database (SwissProt, released on 2014-02-19). The search parameters were the same as above except that methionine oxidation as a search parameter was replaced by phosphorylation of serine, threonine and tyrosine. Only significant phosphopeptide matches exceeding the individual ion scores were considered for further interpretation.

### Bioinformatics

For each of the proteins in the PAZ core proteome we analyzed abundance changes from all experiments. These changes were compiled into a set of protein abundance profiles to identify similar abundance patterns. The abundance profiles were visualized as a line chart where a single profile is represented by a single graph. To identify similar profile patterns, we applied the R-package Mfuzz (Kumar and Futschik, 2007) which implements the fuzzy *c*-means algorithm. Fuzzy clustering assigns each data point to more than one cluster (Pal et al., 1996). Each of the profiles is assigned to every cluster with a membership value in the interval [0, 1]. A membership value of 1 indicates that the profile of a single protein is in full accordance to the corresponding cluster, while a membership value of 0 indicates no accordance. This approach (motivated by Philipp et al., 2013) reduces the influence of noise that is inherent to biological data and, therefore, avoids too stringent selection criteria.

The optimal number of clusters was determined using the Xie-Beni index (Xie and Beni, 1991). This index was computed for a range of partitions of the dataset and indicates the quality of the partition. The lower the value, the better the partition is. The clustering itself was performed in Euclidean space, therefore, the abundance changes had to be standardized to a mean value of zero and a standard deviation of one. This procedure guarantees that vectors of proteins with similar profiles were close in Euclidean space.

Each profile cluster was then functionally analyzed to identify biological processes. For the functional enrichment we assigned proteins to the cluster for which they had a membership value of >0.5. Using the online Panther classification system we identified the enriched biological processes in each cluster. We performed a Panther overrepresentation test with Panther version 10.0, release 20150430. Finally, we assigned a keyword to each cluster to characterize it in the biologically most meaningful way.

### Chemical LTP

Coronal slices with a thickness of 400 μm were prepared using the Campden Microtome 7000smz. The recovered slices were submerged at room temperature in ACSF (125 mM NaCl, 3 mM KCl, 1.25 mM NaH_2_PO_4_, 26 mM NaHCO_3_, 2 mM CaCl_2_, 1 mM MgSO_4_ and 10 mM glucose, bubbled with 95% O_2_ and 5% CO_2_, pH 7.4) for at least 1 h prior transferring them to the interface recording chamber. After another recovery period of 1 h in the interface recording chamber, LTP measurements were started. Synaptic responses were evoked by stimulating Schaffer collaterals between CA3 and CA1 with 100 μs bipolar pulses trough bipolar tungsten electrodes (Microprobes, Gaithersburg, MD, USA). Recording electrodes (6–8 MΩ glass microelectrodes filled with ACSF) were placed in stratum radiatum of CA1 region to measure extracellular excitatory postsynaptic potentials (fEPSPs). Paired-pulse facilitation (PPF) was measured by applying stimuli at an inter-pulse interval of 50 ms before and after running a LTP induction. PPF was expressed as the fEPSP slope of the second response relative to the first. For baseline recordings, synaptic responses were evoked for 20 min every 30 s before LTP induction at a stimulation intensity corresponding to 30–50% of maximum fEPSP response. The stimulation intensity was kept constant over the whole recording duration. To induce LTP, the slices were perfused with ASCF containing 25 mM TEA for 15 min. fEPSPs were amplified and low-pass filtered at 2 kHz using an EXT-10–2F (NPI electronic instruments, Tamm, Germany) amplifier. The traces were sampled at 50 kHz and analyzed using Clampfit (Molecular devices, Sunnyvale, CA, USA). The fEPSP slope corresponds to the rise slope between 20% and 80% of peak amplitude.

### Mitochondrial Activity Determined by Alamar Blue Assay

#### Subcellular Fractionation of Synaptosomes from Mouse Hippocampus

Subcellular fractionation of synaptosomes derived from mouse hippocampus was performed according to the guidelines by Weingarten et al. (2015). The following modifications were applied: Individual hippocampi was dissected from native mouse brain and homogenized in 0.4 mL of preparation buffer (5 mM Tris-HCl, 320 mM sucrose, pH 7.4) containing the protease inhibitors antipain, leupeptin, chymostatin (2 μg/mL each), pepsatin (1 μg/mL) and benzamidine (1 mM). Unless mentioned otherwise the material was kept at 4°C during the entire preparation. The hippocampal homogenate was centrifuged for 2 min at 2800_gav_ (Beckman ultracentrifuge TLX Optima/ rotor no. TLA 120.2). The resulting pellet was discarded and the supernatant was further fractionated by discontinuous Percoll gradient centrifugation. To prepare the Percoll gradient, 1 mL of Percoll solution (3%, 10%, 23% (v/v) in preparation buffer) were consecutively layered on top of each other. The previously collected supernatant was layered on top. Subsequent to centrifugation using the TLA 100.4 rotor for 7 min at 35,000_gav_, fractions containing synaptosomes were collected and diluted twofold in preparation buffer. After centrifugation for 35 min at 50,000_gav_ (rotor no. TLA 100.4) the supernatant was collected for further analysis and the resulting pellet was resuspended in 2 mL Locke’s buffer (154 mM NaCl, 5.6 mM KCl, 2.3 mM CaCl_2_, 1 mM MgCl_2_, 3.6 mM NaHCO_3_, 5 mM glucose, 5 mM HEPES, pH 7.2) followed by centrifugation for 10 min at 4166_gav_ (TLA 100.4 rotor). Subsequently the supernatant (S2) was collected for later analysis and the pellet (P3) was resuspended in 100 μL Locke’s buffer. To quantify the protein content of the synaptosomal fraction the BCA-assay kit (#23225; Pierce, Rockford, IL, USA) was applied.

#### Metabolic Activity of Mitochondria Derived from Hippocampal Synaptosomes (Alamar Blue Assay)

The measurement of Alamar Blue fluorescence in synaptosomal preparations was performed according to the guidelines by Springer et al. (1998). The following modifications were applied: 5 μg of synaptosomal fraction derived from mouse hippocampi were pipetted as triplicates into a 96-well plate. Locke’s buffer was added to reach a final volume of 90 μL. Subsequently Alamar Blue was added as 10% of the final volume. After an incubation time of 0.5 h, 1 h and 15 h at 37°C, the fluorescence was measured using the Tecan Infinite^®^ M200 Pro (excitation/emission: 530/590 nm). For analysis of background activity, samples containing equal amounts of synaptosomal protein that had been heated to 95°C for 5 min prior to the addition of Alamar Blue were used.

#### Inhibition of Mitochondrial Function

To demonstrate that changes in Alamar Blue fluorescence directly correlate with mitochondrial function in the synaptosomal preparation, an additional incubation with the mitochondrial complex I inhibitor Rotenone was performed. Equal amounts of synaptosomes (5 μg) were incubated with Rotenone (0.25 μM, 2.5 μM and 25 μM) for 20 min at 37°C. After the incubation, Alamar Blue was added (10% final volume) and an additional incubation for 15 h at 37°C was performed. Subsequently fluorescence was measured using the Tecan Infinite^®^ M200 Pro (excitation/emission 530/590 nm).

## Results

We previously identified APP as a constituent of the PAZ (Laßek et al., 2013) and reported severe changes in abundance of key proteins at the PAZ upon APP-deletion (Laßek et al., 2014). Furthermore, we demonstrated that APP is embedded into the evolutionary conserved active zone protein complex, comprising ELKS, CASK, bassoon, RIM and Munc18. The integration of APP into the hippocampal PAZ proteome network revealed its context-sensitive characteristics and crucial role in presynaptic physiology (Laßek et al., 2016). Based on these novel insights, we addressed the question how loss of APP affects the hippocampal PAZ proteome during aging. We designed our study with a special emphasis on LTP stimulation and metabolic activity in young and old APP-KO mice.

**Figure 1:**
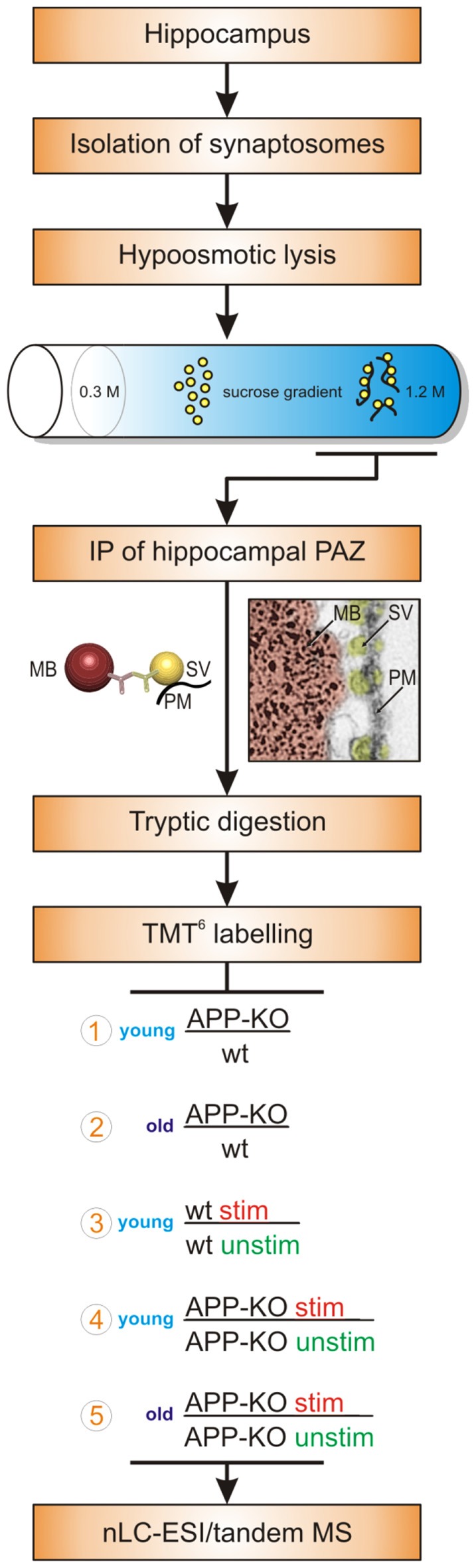
**Workflow.** The workflow illustrates the purification strategy (subcellular fractionation and immunopurification, IP) of the hippocampal presynaptic active zone (PAZ) and the quantitative proteomic approach (tandem mass tag, TMT^6^). Moreover, biological conditions chosen for the individual experimental setups are depicted and encircled with numbers 1–5. Abbreviations: MB, magnetic bead; MS, mass spectrometry; nLC_ESI, nano liquid chromatography electrospray ionization; PM, plasma membrane; stim, stimulated; SV, synaptic vesicle; unstim, unstimulated; wt, wildtype.

We isolated the native hippocampal PAZ from age matched young and old individual mice employing subcellular fractionation and IP (Figure 1). Importantly, our target protein for IP, the synaptic vesicle protein2 (SV2), revealed no changes in abundance in all experiments. Our isobaric labeling approach (tandem mass tag, TMT6) combined with high-resolution mass spectrometry has turned out to be well suited for comparing protein abundance between two or more biological conditions (Laßek et al., 2016). Briefly, the abundance of reporter tags reflects the ratio of peptides in biological triplicates and was used for quantitative analysis (Figure 1). Proteins were considered to be significantly changed in abundance if they reach a threshold of ±20%. We defined our common core proteome (419 proteins) for the hippocampal PAZ by including only those proteins being significantly identified in all experiments (*n* = 15) and animals (*n* = 30). For each condition six animals (3/3) were used. In our approach, postsynaptic proteins, including proteins associated with the postsynaptic density, were almost absent after purification. The experiments comprise the analysis of young APP-KO as compared to wt (encircled 1 in the workflow, Figure 1), old APP-KO as compared to wt (encircled 2 in the workflow, Figure 1), stimulated young wt as compared to unstimulated young wt (encircled 3 in the workflow, Figure 1), stimulated young APP-KO as compared to unstimulated young APP-KO (encircled 4 in the workflow, Figure 1) and finally stimulated old APP-KO as compared to unstimulated APP-KO (encircled 5 in the workflow, Figure 1). The terms stimulated and unstimulated refer to the LTP experiment (for details see “Materials and Methods” section) performed on hippocampal slices derived from young and old wt and APP-KO mice.

### Changes in Protein Abundance between Young and Old APP-KO Mice

Previously, analysis of protein abundance in young and old APP-KO mice as compared to their respective wt mice revealed remarkable differences. However, affected proteins differ in young APP-KO as compared to old mutants (Supplementary Data Sheet 1, Core Protein abundance). The abundance of calmodulin that was severely reduced to 31% in young APP-KO mice as compared to wt (100%) returned to normal levels in old APP-KO (113%) as compared to the respective wt animal. Similarly, downregulation of clathrin-light chain A (70%), the gamma subunit of trimeric G proteins (37%), synaptogyrin-1 (71%), the subunit d1 (63%) of the V-type proton ATPase, as well as neuromodulin (79%) in young animals was restored to wt levels in old APP-KO (Supplementary Data Sheet 1, Core Proteome abundance).

In contrast, the fusion clamp complexin-1 (62%) and the voltage-dependent anion-selective channel proteins (VDACs, 70%-76%) were downregulated in old APP-KO as compared to young mutants. Subunits of the actin-associated motor myosin light (143%) and heavy chain (144%), neurofilament medium (126%) and heavy chain (130%), reticulon-1 (121%), subunits D (121%) and H (121%) of the v-type proton ATPase and the synaptic vesicle protein VAMP-2 (133%) were upregulated in old APP-KO. Whereas the tubulin alpha-1A (121%) and 4A (129%) chains were upregulated tubulin beta-2A (73%) was downregulated in old APP-KO. Surprisingly, a large variety of mitochondrial proteins are downregulated in elderly mutants (Supplementary Data Sheet 1, Core Proteome abundance).

### LTP Induction and Changes in the Phosphorylation Status of Proteins upon Stimulation

APP deletion accounts for impairments in learning and memory upon aging and electrophysiological recordings have already visualized a decrease in LTP for old APP-KO mice (Phinney et al., 1999; Ring et al., 2007). We addressed the question whether LTP induction has an impact on the hippocampal PAZ proteome derived from young and old APP-KO mice. We chose this approach for the following reason: the chemical induction of LTP in hippocampal slices was performed under physiological conditions to retain basic cellular properties. This includes not only the ability to set up and maintain membrane potential, but also to synthesize ATP and allow protein kinases to act inside the nerve terminal.

**Figure 2:**
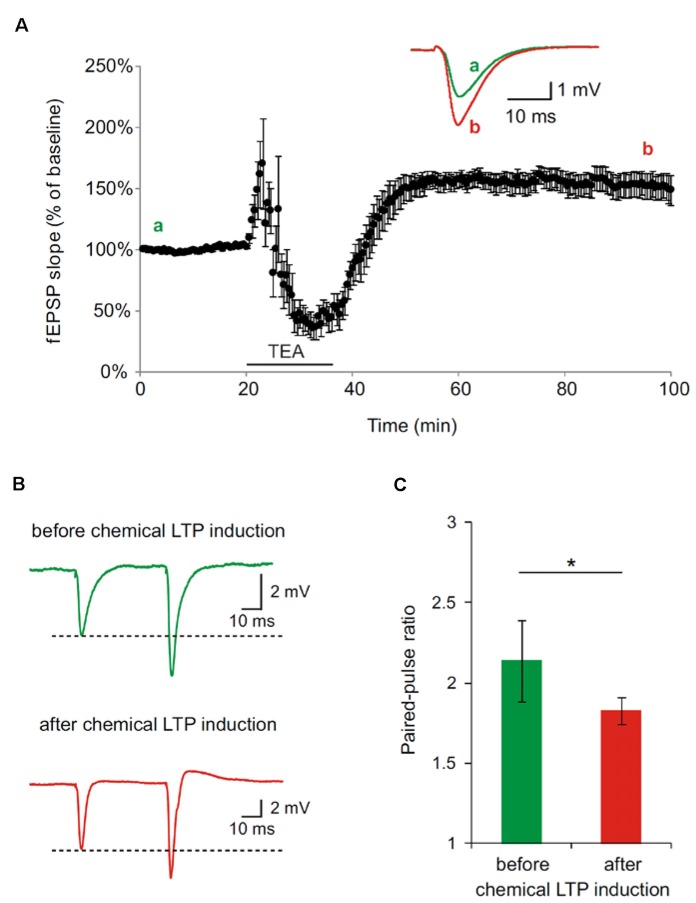
**Paired-pulse facilitation (PPF) is decreased after tetraethylammonium (TEA)-induced potentiation in hippocampal slices of 4 month old wildtype (wt) mice. (A)** Long-term-potentiation (LTP) was chemically induced by bath perfusion of 25 mM TEA-Cl in ACSF. Inset shows example traces before (a) and after (b) induction of LTP with TEA (stimulus artifacts were removed). **(B)** Representative traces of PPF measurements before and after TEA-induced LTP (stimulus artifacts were removed). **(C)** Paired-pulse ratio (calculated from fEPSP slope) at an inter-pulse interval of 50 ms is significantly reduced after the induction of chemical LTP with TEA (SEM, *p < 0.05, Wilcoxon Signed-Rank Test).

In the first set of experiments, we generally demonstrated in hippocampal slices, derived from wt mice, that the applied protocol to chemically induce LTP leads to a potentiation of synapses as shown by an increase in the fEPSP slope (Figure 2). Further, the synaptic potentiation by bath perfusion of 25 mM TEA-Cl in ACSF involves presynaptic changes. PPF is decreased after TEA-induced LTP in hippocampal slices indicated an increase in presynaptic vesicle release probability (*p < 0.05; Figure 2A). Representative traces of paired-pulse-facilitation before (a, green) and after (b, red) LTP induction are highlighted as curves (Figure 2B) and bar diagram (Figure 2C).

Next, we induced chemical LTP in hippocampal slices derived from young and old APP-KO mice, purified the hippocampal PAZ, as described in the methods section, and performed quantitative proteomics. It is remarkable, that virtually all PAZ players remained unaltered, i.e., they did not reach the threshold of ±20%. Key proteins involved in neurotransmitter release like the SNARE proteins SNAP25, syntaxin-1 and VAMP-2 or other prominent synaptic vesicle proteins such as SV2 and synptotagmin-1 revealed no changes in protein abundance. The same applies to proteins involved in calcium homeostasis like calmodulin, neuromodulin and L-type calcium channels or proteins involved in intracellular signaling such as CaMKII, 14-3-3 and PKC (Supplementary Data Sheet 2). However, at the phosphoprotein-level, we could detect upregulation for important players in intracellular signaling at the PAZ (Supplementary Data Sheet 2).

The induction of LTP accounts for rapid changes in the phosphorylation status of proteins rather than changes in the expression pattern. Therefore, we could unravel a variety of signaling and calcium-dependent proteins that reveal remarkable changes in their phosphorylation degree both of serine/threonine or tyrosine (S/T or Y) amino acid residues in old APP-KO mice (mean highest percentage of phosphorylation as compared to young APP-KO set to 100%). Phosphoprotein candidates of our core proteome that underwent stronger stimulation-dependent changes in old APP-KO comprise members of the 14-3-3 family, subunits of the sodium/potassium-transporting ATPase, neuromodulin, syntaxin-1B, VDAC1, rabconnectin-3, synaptophysin, three subunits of PKC and the alpha subunit of CaMKII. The two isoforms gamma and zeta/delta of the 14-3-3 (137% or 144% respectively) adaptor molecules involved in synaptic plasticity revealed a significantly higher phosphorylation degree in stimulated old APP-KO as compared to stimulated young APP-KO. Phosphorylation of 2′,3′-cyclic nucleoside monophosphate phosphodiesterase (CNP, 194%) an indicator of aged membranes is almost twofold higher in the hippocampal PAZ of stimulated old APP-KO than in young animals. Two subunits (ß1, 137% and α3, 152%) of the sodium/potassium-transporting ATPase are significantly higher phosphorylated in several phosphopeptides in elderly mice (Supplementary Data Sheet 2). Similarly, the PKC substrate and calmodulin binding protein neuromodulin (139%) is more strongly phosphorylated in old stimulated mice. Moreover, syntaxin-1B (127%) involved in synaptic vesicle docking/priming and neurotransmitter secretion revealed a higher phosphorylation degree in the old animals. VDAC1 (173%) also involved in synaptic transmission and learning is strongly phosphorylated in old stimulated APP-KOs. In addition, rabconnectin-3 (139%), a key controller of neuronal homeostatic processes and the synaptic vesicle protein synaptophysin (125%) are also more strongly phosphorylated in elderly mutants. Three subunits (alpha, beta and gamma up to 165%) of PKC revealed a high degree of phosphorylation. The key protein in LTP CaMKII (162%) is hyperphosphorylated in the hippocampal PAZ of old stimulated mice (Supplementary Data Sheet 2). Moreover several mitochondrial proteins are more strongly phosphorylated in old stimulated as compared to young stimulated mice: aspartate aminotransferase (137%), a subunit of ATP synthase (153%), malate dehydrogenase (157%) and aconitate hydratase (143%).

#### Fuzzy Clustering

**Figure 3:**
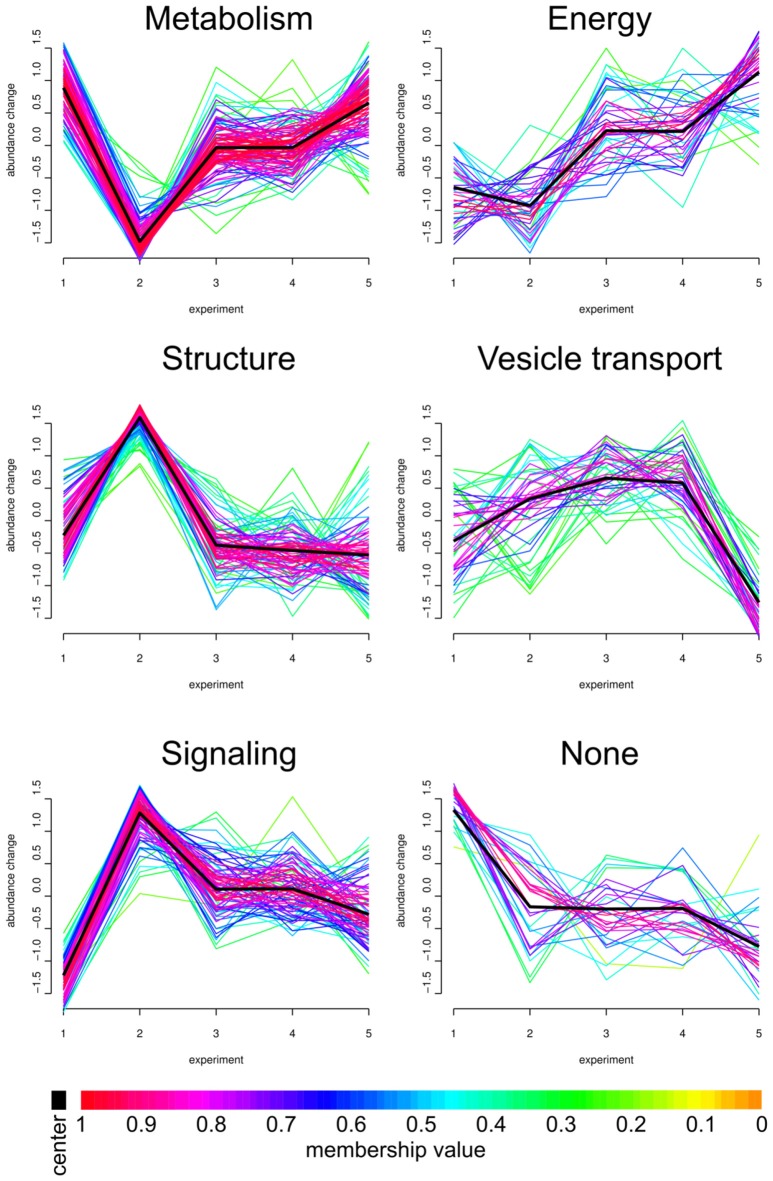
**Fuzzy clustering.** We analyzed our core proteome employing Fuzzy clustering. Here, changes in abundance profile of individual proteins (for all experiments; *x*-axis) are graphically represented. Individual abundance progressions (*y*-axis) were grouped and sorted by functions and structures. The individual Fuzzy clusters are divided into five categories: Metabolism, Energy, Structure, Vesicle transport and Signaling. APP-KO young/wt young (1, number as in Figure 1), APP-KO old/wt old (2), wt young stimulated/unstimulated (3), APP young stimulated/unstimulated (4) and APP-KO old stimulated/unstimulated (5). Proteins grouped in the six clusters are provided in Supplementary material as Panther database analysis (Supplementary Data Sheet 3).

The impact of APP deletion upon aging and stimulation revealed a large variety of individual proteins being significantly up- or downregulated at the native hippocampal PAZ. Based on the Xie-Beni index, the core proteome was grouped in six so called fuzzy clusters (Figure 3). The Xie-Beni index for a partition of the dataset into six clusters was 0.00196. A partition of the dataset into two clusters gave a smaller index of 0.00065 but led to an insufficient discrimination of abundance profiles. The fuzzy *c*-means algorithm allows for the graphic representation of similarities in protein abundance changes over different biological conditions.

The PAZ core proteins were assigned to the six clusters with the abundance change shown on the *y*-axis and the different biological conditions on the *x*-axis. As highlighted in the workflow (Figure 1), the numbers on the *x-axis* indicate individual experiments and encompass the following conditions: (1) APP-KO young/wt young; (2) APP-KO old/wt old; (3) young wt simulated/unstimulated; (4) APP-KO young stimulated/unstimulated and (5) APP-KO old stimulated/unstimulated. The individual fuzzy clusters are divided into five categories: Metabolism, Energy, Structure, Vesicle transport and Signaling. The average trend for each cluster is highlighted as a black line. The color gradient reflects the deviation between the average pattern and individual curve progression within each cluster. The comparison between (1) young APP-KO and (2) old APP-KO revealed drastic changes in abundance within the metabolic, structure and signaling cluster, whereas energy and vesicle transport clusters remained almost unaltered. The abundance of proteins involved in metabolism are downregulated, whereas proteins involved in the structure of the PAZ or involved in signaling are upregulated in old APP-KO as compared to young mutants. Interestingly, stimulation of (3) young wt and (4) APP-KO depicted a consistent curve progression without any slope within each cluster. The comparison of stimulated (4) young and (5) old APP-KO demonstrated a rise in the curve progression for the clusters metabolism and energy, whereas vesicle transport proteins are downregulated. Interestingly, the majority of proteins within these clusters belongs to mitochondria and point to an imbalance in the bioenergetics system at neurotransmitter release sites in old APP- mutants. The heterogeneous none-group comprises proteins that could not be assigned to a distinct group by the fuzzy c-means algorithm, because their abundance profile (over all experiments) was not in line with the computer generated one.

**Figure 4:**
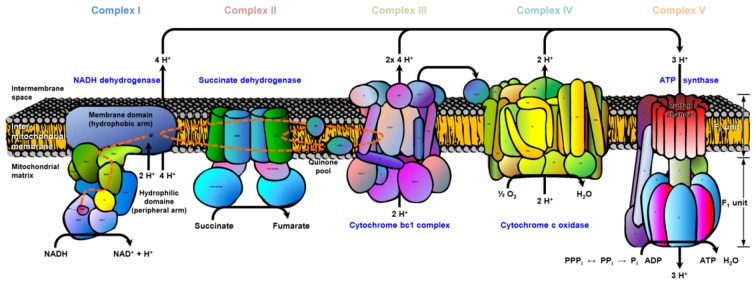
**Electron transport chain (ETC).** The cartoon illustrates the ETC complexes (I–V) and affected subunits. The register provided in supplementary material (Supplementary Data Sheet 4) illustrates the gene names for each ETC complex (I–V) and affected subunits (highlighted in red). Data are exemplarily depicted for old APP-KO mice. The overall pathway mapping is based on kyoto encyclopedia of genes and genomes (KEGG) database (Release 78.0, April 1, 2016).

We illustrated exemplary the ETC for old APP-KO highlighting all proteins with significant changes (mainly downregulation) in abundance based on the Kyoto Encyclopedia of Genes and Genomes (KEGG) pathway mapping (Figure 4). The ETC consists of five complexes with specific enzymatic electron donors and acceptors. Almost half of the core subunits of NADH dehydrogenase (complex I) display changes in protein abundance (highlighted in red). Complex I is basically responsible for the transfer of electrons from NADH to the respiratory chain, with ubiquinone acting as electron acceptor. Comparing the affected core subunits of complex I indicate that the majority is not essential for the minimal assembly of catalytic activity (NADH dehydrogenase [ubiquinone] iron-sulfur protein 4 Ndufs4, 6; Ndufa2, 5, 6, 9, 10, 12, 13; Ndufb 3, 4, 5, 10; Ndufc2). However, six core subunits are involved in catalysis and revealed significant changes (Ndufs1-3, 7; Ndufv1-2). For complex II, the major catalytic subunit succinate dehydrogenase complex subunit A (SDHA) as well as the directly connected iron sulfur protein (SDHB) is affected by APP-deletion. For Complex III, (cytochrome bc1 or the ubiquinol-cytochrome c reductase, respectively) both core subunits (COR1, QCR2) required for the assembly of the complex reveal changes in abundance. Complex IV, also named cytochrome-c oxidase, catalyzes the reduction of oxygen to water and is the terminal electron acceptor of the respiratory chain. The functional core of complex IV is formed by core subunits 1-3 (COX1-3). Deletion of APP accounts for changes in protein abundance for core subunit 2 (COX2). COX2 is responsible for the transfer of electrons from cytochrome c to the bimetallic center of the catalytic subunit 1. Moreover, COX6b, implicated in the connection of COX monomers into their physiological form, is also affected by the deletion. The ETC is finally completed by complex V, also named F-type ATPase. F-ATPases are divided into the membrane integral FO domain, containing the proton channel, and the peripheral, extramembraneous catalytic core F1. Both subunits, alpha and beta, form the catalytic F1 core and reveal significant changes in protein abundance. The gamma subunit, acting as rotary motor protrudes into the alpha/beta core, is also affected by the deletion of APP. The proton channel, divided into nine subunits (a-g, F6, F8) reveals changes in abundance for more than half of its constituents (b, d, e, g, F6). Subunit b, d and F6 are involved in building up the peripheral stalk that connects F1 and FO and prevent the unintentional rotation of other subunits with the rotary motor. Subunit e is involved in structural features, like dimerization of ATP synthase or stabilization of subunit g, whose function is already unknown.

#### Metabolic Activity

The differences in protein abundance for each ETC complex point to alterations in catalytic activity and structural integrity. In this context, we addressed the question, whether APP-KO accounts for such a physiological effect on mitochondria at neurotransmitter release sites. The most common and suited method to analyze mitochondrial metabolic activity is the Alamar Blue assay. Resazurin, the fluorometric indicator of metabolic activity, has a redox potential of *E* = +380 mV and can therefore not become an intermediate until the terminal electron transfer step (the final reduction of oxygen to water at complex IV). Therefore, resazurin does not impair the electron transport and provides a high sensitive indication of mitochondrial activity.

**Figure 5:**
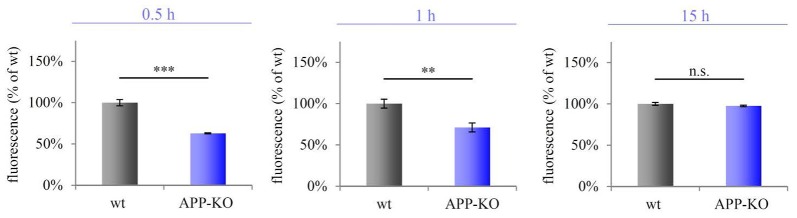
**Impaired metabolic activity in old APP-KO.** APP-KO has an impact on mitochondrial metabolic activity at hippocampal PAZs. The percentage difference (Δ) in fluorescence was calculated between wt and APP-KO and set to 100% for wt ± SEM (fluorescence % of wt). Equal volumes (100 μL) were loaded per well. Incubation for 0.5 h: Δ = 41%; ***p < 0.001; 1 h: Δ = 37%; **p < 0.01, 15 h: Δ = 1%; n. s. Note, after 15 h saturation of resazurin conversion is reached. Statistical analysis was performed employing the unpaired Student’s *t*-test: ***p < 0.001, **p < 0.01, n. s. not significant, *n* = 3 animals with technical triplicates each.

We purified hippocampal synaptosomes derived from aged wt and APP-KO mice employing discontinuous Percoll gradient centrifugation. To check for mitochondrial metabolic activity, we incubated synaptosomes with Alamar Blue and fluorescence levels were measured after 0.5 h, 1 h and 15 h. The percentage difference (Δ) in fluorescence was calculated between wt and APP-KO (fluorescence % of wt 100%, ± SEM, *n* = 3 animals with technical triplicates each). Equal volumes (100 μL) were loaded per well. After 0.5 h Alamar Blue fluorescence was decreased by 59% for APP-KO as compared to wt (100%; Δ = 41%; ***p < 0.001; Figure 5). Incubation for 1 h still revealed a striking divergence in fluorescence level for wt and APP-KO (Δ = 37%; **p < 0.01; Figure 5). However, after 15 h Alamar Blue fluorescence values obtained for APP-KO mice were comparable to those obtained for wt mice (Δ = 1%; n. s.; Figure 5). The data obtained for Alamar Blue fluorescence point to an effect of APP-deletion on mitochondrial metabolic activity in hippocampal synaptosomes. Additionally, we performed selective inhibition of complex I using Rotenone (Supplementary Image 1) and compared again APP-KO and wt. As described above, the iron-sulfur protein (Ndufs2) of complex I, critically involved in the electron transfer to ubiquinone, is affected by APP-deletion. Rotenone specifically inhibits this transfer and accounts for significant reduction in Alamar Blue fluorescence.

## Discussion

Within this study, we could demonstrate that APP deletion has an impact on the hippocampal PAZ proteome during aging, accompanied by a severe decrease in metabolic activity. Notably, LTP induction accounts for alterations within the phosphorylation level of prominent signaling molecules and kinases—particularly the CaMKII the key protein involved in LTP, together with PKC and the 14-3-3 proteins are hyperphosphorylated in old APP-KO. Previously, we reported that APP executes regulatory functions within the synaptic vesicle cycle, cytoskeletal rearrangements and Ca^2+^-homeostasis. Moreover, we observed significant downregulation of the mitochondrial ATP synthase-coupling factor 6 and subunit e in young APP-KO mice (Laßek et al., 2016). Despite these changes, all subnetworks within the hippocampal PAZ persisted and maintained functionality (Laßek et al., 2016). This is in accordance with the presumed compensatory mechanisms acting in young APP-KO mice preventing the onset of cognitive decline as observed in the elderly mice (Phinney et al., 1999; Priller et al., 2006; Laßek et al., 2016).

Neurons demand high amounts of energy for orchestrated action of neurotransmission, up- and downstream signaling and synaptic plasticity (Boveris and Navarro, 2008). In this context, the most important guarantors for sufficient energy metabolism, calcium- and redox homeostasis are mitochondria (Yin et al., 2014; Grimm et al., 2016). The progression of age-dependent diseases might be accompanied by the improper response of neurons to the reduced metabolic rates and decline in cognitive performance is associated with decrease in bioenergetics metabolism (Swerdlow, 2007; Yin et al., 2014). AD is closely related to mitochondrial dysfunction which affects APP expression, enzymatic processing and an increase in Aβ production (Gabuzda et al., 1994; Webster et al., 1998). The decrease in energy status, paralleled by changes in the redox-state and increase in ROS, are features of aging and neurodegeneration. Mitochondria have a variety of functions: they support the intracellular energy demand by producing ATP, affect redox-sensitive kinases via second messengers H_2_O_2_ and NO and regulate the NAD^+^/NADH homeostasis, involved in maintenance of mitochondrial energy statues (Yin et al., 2014). Deletion of APP accounts for drastic changes in mitochondrial protein abundance at hippocampal neurotransmitter release sites. These proteins have in common that they either contribute to the assembly of the respective enzymatic complex or trigger their catalytic function. Our results indicated that old APP-KO mice display a dysregulation in bioenergetics metabolism that can contribute to the onset of impairments in learning and memory formation.

Since mitochondrial dysfunction and cognitive decline are characteristic for the progression of AD, it was hypothesized that AD is a metabolic disease, initiated by age-related mitochondrial deficiency (Leuner et al., 2012a,b). Decline in enzymatic activity during aging is a consequence of the thermodynamic instability of large biomolecules. Especially neurons depend on the capacity to convert energy from the external environment e.g., glucose and lactate, for their biosynthetic work (ATP production). It was suggested that the upregulation of oxidative phosphorylation (OXPHOS) acts as a compensatory mechanism to sustain proper energy production and viability of impaired neurons. The concept of an inverse Warburg effect assumes that there will first be an increase in OXPHOS, followed by competition between healthy and affected neurons for oxidative energy substrates (e.g., glucose) and finally the transformation of formerly healthy neurons into bioenergetically affected ones (Demetrius et al., 2014).

Oxidative phosphorylation as well as protein phosphorylation are basic mechanistic steps for energy production. Under physiological conditions they provide a proper energy supply of the aging brain but culminate in neurodegeneration during the pathogenesis of AD (Demetrius et al., 2014).

Among the most suitable and frequently used experimental approach to address alterations in synaptic plasticity are electrophysiological recordings (LTP recordings). The impact of APP deletion on the induction and maintenance of LTP in hippocampal neurons has thus far only been discussed under postsynaptic aspects (Ring et al., 2007). Interestingly, we could observe drastic changes in the phosphoprotein level in old APP-KO mice after induction of LTP. As previously described, APP-KO mice develop deficits in learning and memory only upon aging (Ring et al., 2007). It was suggested that the loss of APP is compensated in younger animals by the increased number of hippocampal synapses (Priller et al., 2006). However, this increase in synapse number did not continue into old age (Phinney et al., 1999; Priller et al., 2006).

As APP is a member of a small gene family including in mice the APP-like proteins APLP1 and APLP2 it appears likely that the APLPs may compensate the loss of APP in young mice. Consistent with partially overlapping functions, recently generated conditional APP/APLP2 double knockout mice revealed reduced synaptic density, impaired branching, deficits in LTP and hippocampus dependent tasks already in young mice (Hick et al., 2015). The functional integration of APP into the hippocampal PAZ proteome supported the notion of a physiological role of APP in synaptic transmission and plasticity (Laßek et al., 2016).

Upon aging APP-KO mice develop not only impairments in LTP (Dawson et al., 1999; Seabrook et al., 1999; Ring et al., 2007) but these deficits are also associated with age-dependent impairments in behavior such as conditioned avoidance of an electric shock and deficits in spatial learning as assessed by the Morris water maze (Dawson et al., 1999; Ring et al., 2007). Until now the molecular mechanism underlying the LTP deficit remains elusive.

LTP induction induces rapid changes at the level of protein phosphorylation rather than at the translational level. Our study revealed an increase in the phosphorylation of prominent signaling molecules CaMKII, 14-3-3 and PKC only in old APP-KO mice upon LTP induction. At the neuromuscular junction in *Drosophila* the 14-3-3 protein named Leonardo is essential for transmission augmentation and post-tetanic potentiation enabling learning (Broadie et al., 1997). It has been reported, that 14-3-3 proteins are important regulators of presynaptic Ca_v_2.2 channel activities and through this mechanism may contribute to the regulation of synaptic transmission and plasticity (Li et al., 2006). Neuronal CaMKII regulates several important neuronal functions, including neurotransmitter synthesis, neurotransmitter release, modulation of ion channel activity, cellular transport, cell morphology and neurite extension, synaptic plasticity, learning and memory (reviewed in Yamauchi, 2005). Decreased Phospho-CaMKII levels have been suggested to account for the stress-induced reduction in hippocampal LTP expression (Gerges et al., 2004). Employing *in vivo* conditional protein knockout of CaMKII, Wang et al. (2003) reported that a precise level of CaMKII reactivation is essential for the consolidation of long-term memories in the brain. It is tempting to speculate that during the induction of LTP CaMKII and PKC, both serine/threonine specific kinases, become over-activated through hyperphosphorylation in APP-KO mice, which has a negative impact on synaptic plasticity, and prevents proper learning and memory consolidation. Recently, over-activation of CaMKII was described in hippocampal neurons following synaptic stimulation and increased intracellular Ca^2+^-levels. As a kind of protective mechanism, CaMKII is able to form clusters (spherical clusters, identical in size and shape) preventing excessive protein phosphorylation, independent of the autocatalytic center, due to an imbalance in Ca^2+^-homeostasis (Dosemeci et al., 2007). Additionally, PKC, also an important player in the regulation of synaptic plasticity, causes neurodegeneration upon over-activation (Mattson et al., 1991; Birnbaum et al., 2004).

In this context, we suggest, that cognitive impairments in old APP-KO mice might be associated with an imbalanced phosphorylation-activity of the serine/threonine-specific kinases CaMKII and PKC. Moreover, age-dependent changes in the bioenergetic metabolism at hippocampal neurotransmitter release sites can provide a missing link in unraveling the mechanisms of age-dependent impairment in learning and memory. In summary, our data imply a pole position for APP in learning and memory consolidation and emphasize its progressive regulatory role during aging.

## Author Contributions

ML, JW and WV conceived and designed the experiments. ML, JW, BB, MN, TNA and EH performed the experiments. ML, JW, MW, BB, EH, MN, VG and WV analyzed the data. JA, AA-P, IK, UCM and MK contributed reagents/materials/analysis tools. ML, JW, MW, BB and WV wrote the article.

## Funding

The financial support was provided by the Deutsche Forschungsgemeinschaft (MU 1457/8-1; 1457/9-1 to UCM) and by grants from the Cluster of Excellence EXC 115 and Gutenberg Research College (GRC) University Mainz (to AA-P). JA and IK are partly supported by the Cluster of Excellence “Macromolecular Complexes” Frankfurt am Main. The funders had no role in study design, data collection and analysis, decision to publish or preparation of the manuscript.

## Conflict of Interest Statement

The authors declare that the research was conducted in the absence of any commercial or financial relationships that could be construed as a potential conflict of interest.
