# Supplementary figures and images for: APP Deletion Accounts for Age-Dependent Changes in the Bioenergetic Metabolism and in Hyperphosphorylated CaMKII at Stimulated Hippocampal Presynaptic Active Zones
Source: Front Synaptic Neurosci. 2017 Jan 20;9:1. doi: 10.3389/fnsyn.2017.00001 (PMC5247443; doi:10.3389/fnsyn.2017.00001)

Complex I

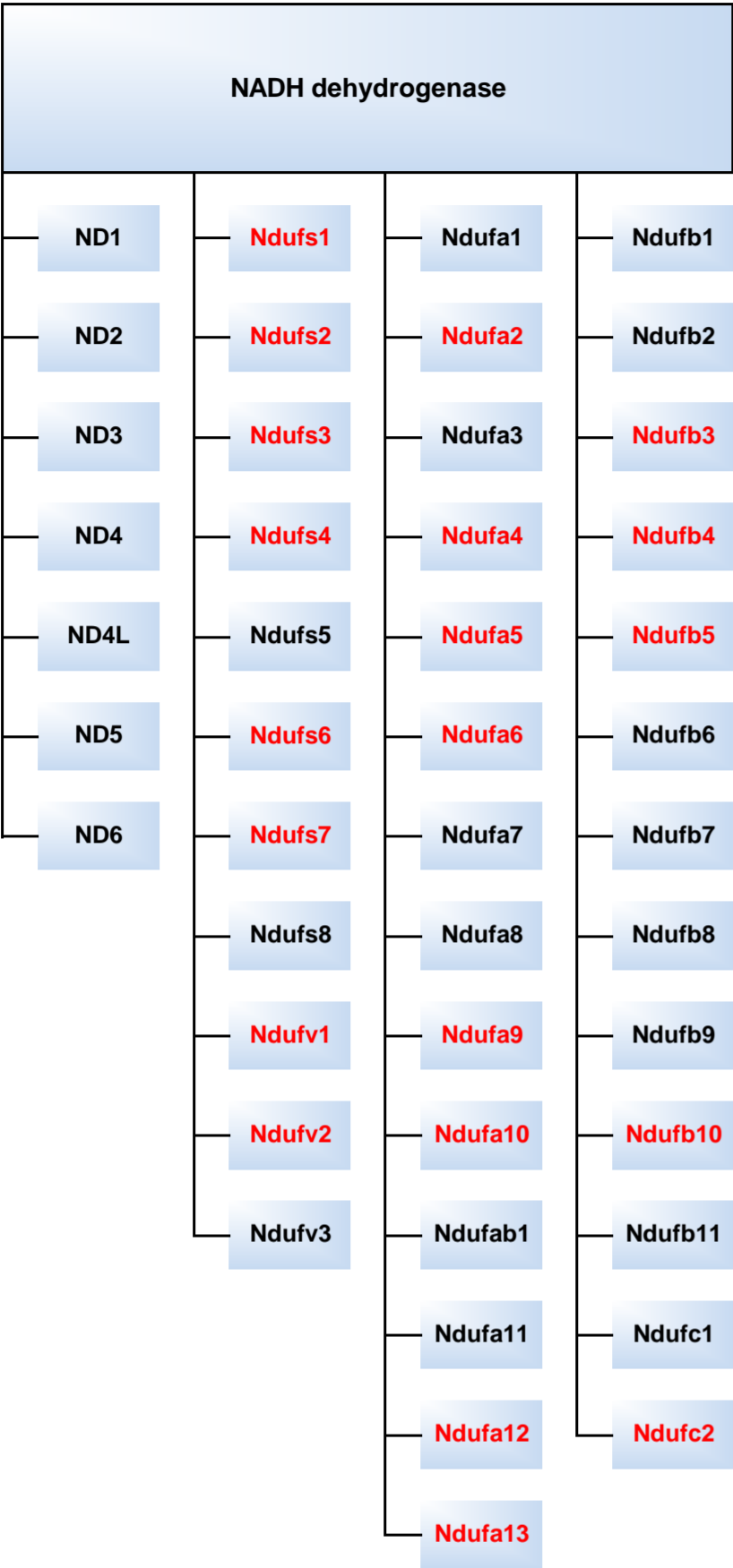

Complex II

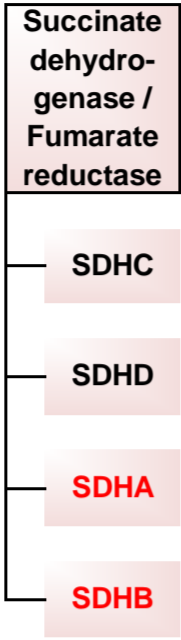

Complex III

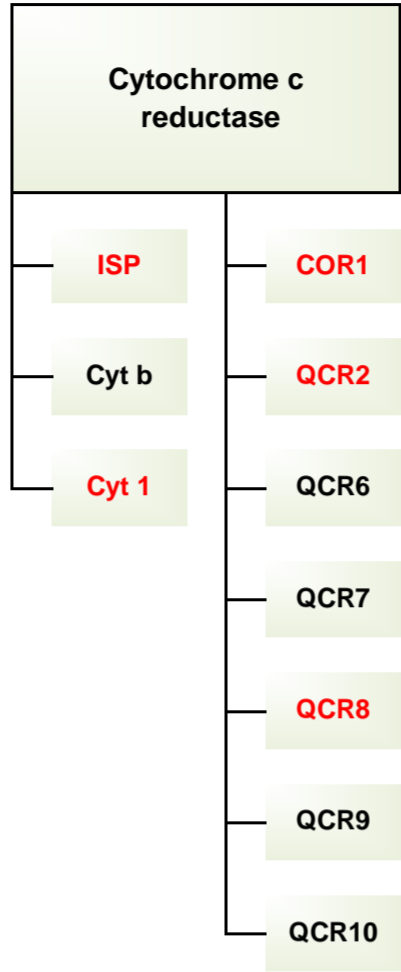

Complex IV

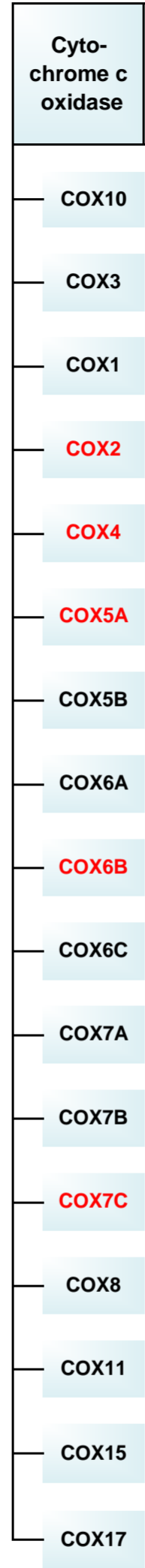

Complex V

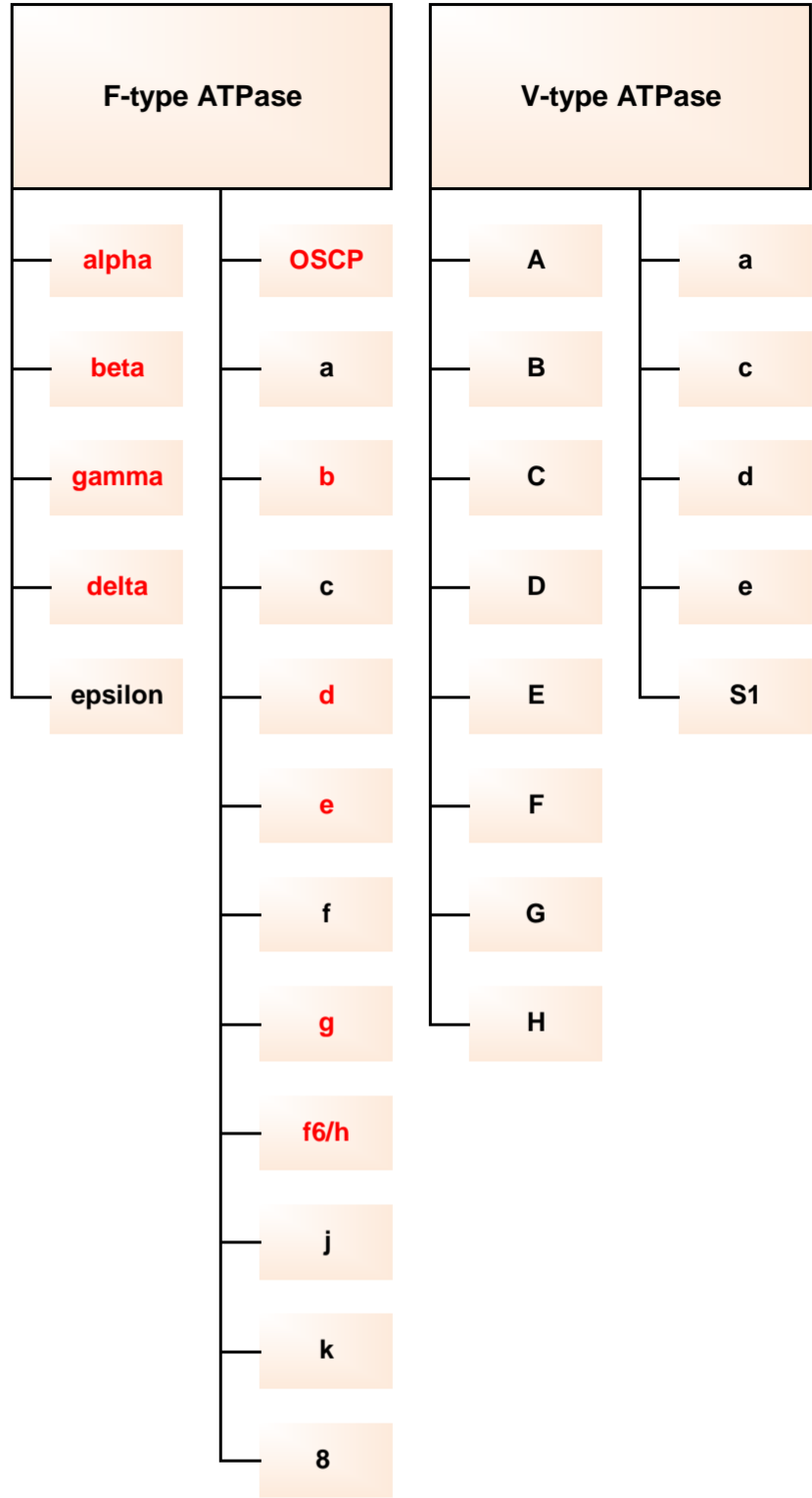

Supplement: Supplementary file 4 [file DataSheet_4.pdf]

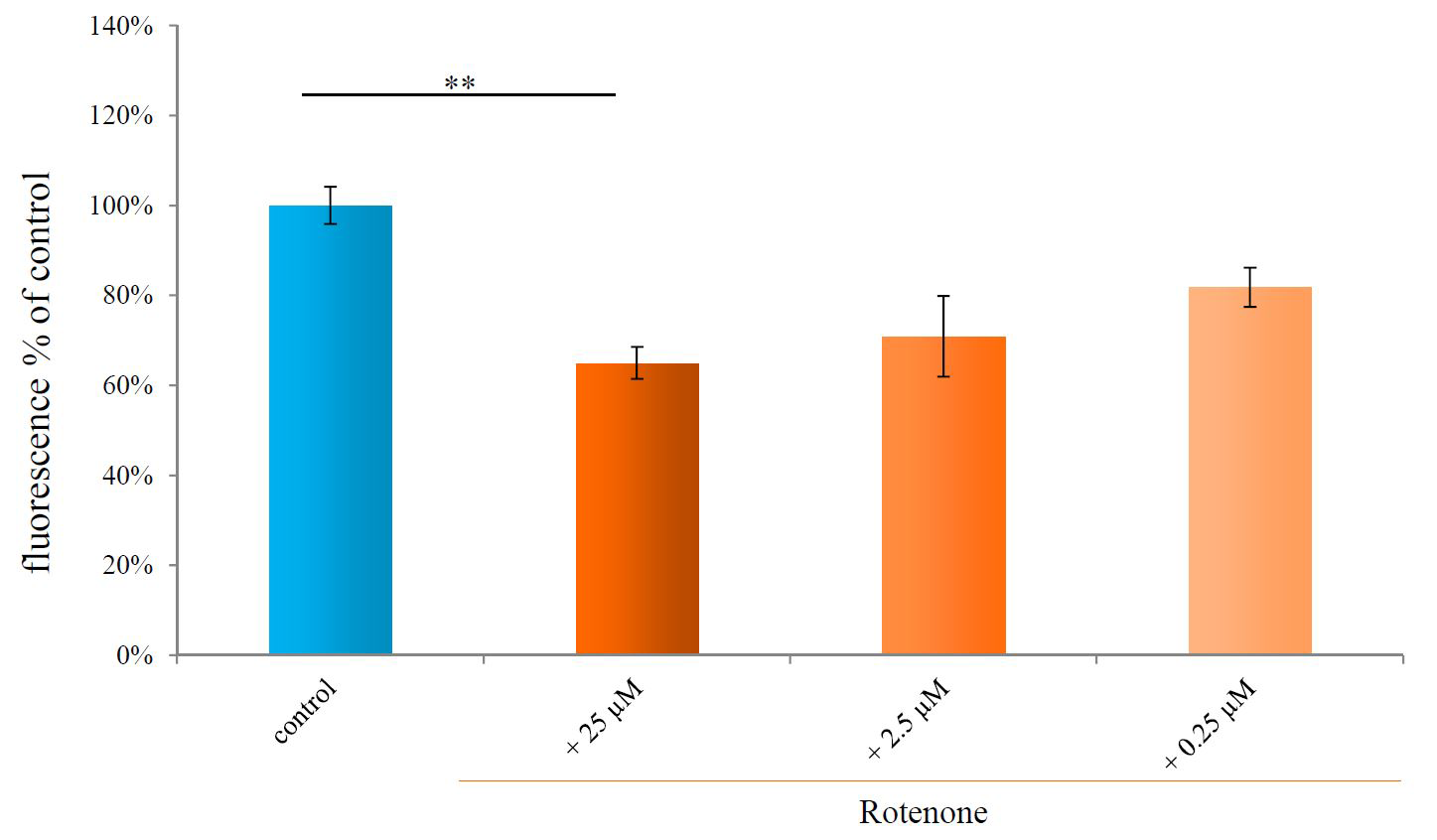

Supplement: Supplementary file 5 [file Image_1.tif]
